# Motif-Cluster: Motif driven prioritization of transcription factor binding clusters

**DOI:** 10.1371/journal.pcbi.1014639

**Published:** 2026-08-05

**Authors:** Mengyuan Zhou, Qiuming Yao

**Affiliations:** School of Computing, University of Nebraska-Lincoln, Lincoln, Nebraska, United States of America; University of Maryland School of Medicine, UNITED STATES OF AMERICA

## Abstract

Genome-wide analyses of transcription factor (TF) motif binding sites have largely emphasized individual high-affinity sites, while overlooking the regulatory importance of locally repetitive motif clusters. Such clusters, including combinations of weak and strong binding sites, can collectively enhance TF occupancy and regulatory activity. Here we present Motif-Cluster, an open-source framework for motif-driven prioritization and visualization of TF binding clusters using sequence information alone. Motif-Cluster integrates a density-based clustering strategy with flexible modeling of binding-site gaps and affinity signals, enabling the identification and ranking of candidate regulatory regions without requiring experimental binding data. Through simulations and multiple real-data analyses, we show that combining gap distributions with binding affinity effectively balances cluster size and signal strength while reducing noise from weak sites. Application to ZNF410 successfully recovers the previously characterized binding clusters in the CHD4 promoter, which are conserved between human and mouse. Additional case studies involving PHB1, TWIST1, and EGR1 further demonstrate the general applicability of the method across diverse transcription factors. Motif-Cluster also provides intuitive visualization and reproducible workflows to facilitate interpretation of spatially dense motif patterns. Overall, Motif-Cluster offers a robust and flexible approach for prioritizing transcription factor regulatory regions from genome-wide motif scans, enabling biological discovery and guiding experimental design, particularly in settings where direct genome-wide binding assays are unavailable.

## Author summary

Genes are controlled by proteins called transcription factors, which bind to specific DNA sequences to turn genes on or off. Traditionally, researchers focus on strong individual binding sites to identify important regulatory regions. However, in several cases reported recently, multiple nearby binding sites, even if individually weak, can work together to regulate gene activity. We developed Motif-Cluster, a computational tool that searches the genome for these clusters of binding sites using DNA sequence information alone. By considering both how closely binding sites are spaced and how strong they are predicted to bind, Motif-Cluster can prioritize regions that are most likely to be biologically important. This tool enables researchers to uncover previously overlooked regulatory elements, particularly in situations where experimental binding data are limited or unavailable. As an open-source and user-friendly tool, Motif-Cluster is broadly applicable and easily adaptable to different transcription factors, organisms, and research needs.

## Introduction

Transcription factors (TFs) regulate gene expression and biological processes by binding to DNA at specific sequence motifs, yet our understanding of how these interactions are encoded and interpreted remains incomplete [1–4]. Most analyses often focus on strong, high-affinity binding sites, but TF recognition in vivo spans a wide spectrum of affinities across the genome, where millions of potential binding sites may exist [5]. Within this landscape, regions exhibiting clusters of nearby binding sites, including both strong and weak motifs, are increasingly recognized as important regulatory units. Such repetitive TF binding sites can create locally elevated concentrations of TFs, allowing even low-affinity interactions to contribute to functional regulation [6]. Experimental work has shown that clusters of low-affinity sites can achieve substantial TF occupancy and drive gene activation in vitro [7], challenging models that prioritize only high-affinity targets. Recent theoretical and empirical studies also reveal that weak clustering can optimize information transfer between molecular signals and downstream gene expression programs, further supporting the biological relevance of spatially clustered, low-affinity binding events [7,8]. Together, these findings highlight the dynamic interplay between binding affinity, spatial context, and regulatory function, motivating the need for computational methods that can systematically identify and prioritize motif clusters from genomic sequences.

While there exists some hypothetical understanding of gene regulatory sequences often comprising clusters of proximal TF binding sites [6,9,10], conducting in vivo studies to further explore and validate these clusters poses a considerable challenge in research endeavors. While ChIP-seq stands as a powerful technique for exploring the genome-wide distributions of chromatin-binding proteins, its efficacy relies on the availability of high-affinity antibodies [11]. Consequently, it may not effectively identify binding sites with lower affinity. While Cut and Run presents a more adaptable method for identifying chromatin-binding proteins [12], it has its own set of challenges and limitations [12]. PADIT-seq is a high-resolution in vitro binding assay capable of detecting hundreds of previously unrecognized lower-affinity transcription factor binding sites [7]; however, applying this technique at genome-wide scale with comprehensive sequence coverage and across diverse transcription factors remains difficult. Among the limited available examples, ZNF410 provides a compelling case of functional binding clusters within the CHD4 promoter that regulate gamma-globin repressors [13]. ZNF410 directly activates the NuRD component CHD4 in human erythroid cells, and this specificity is driven by two highly conserved binding site clusters shared between mouse and human [13–16]. These clusters were independently validated by ChIP-seq [14], cut-and-run assays [13], and our motif analysis [13]. The clear phenotypic impact and well-defined cluster architecture of ZNF410 strongly motivate the development of a general computational tool on the sequence basis.

A sequence and motif based strategy offers a practical and scalable alternative for identifying regulatory regions, especially given that ChIP-seq and cut-and-run data are not universally available across all transcription factors, organisms, tissues, and cell types. TF sequence motifs are broadly available through resources like JASPAR [17] or HOCOMOCO [18]. Subsequently, putative binding sites spanning the genome or chromosome can be computed using FIMO motif scanning [19], or alternatively, obtained from pre-calculated files via the PWMScan website [20]. Sequence motifs enable the identification of binding sites spanning a wide range of affinities, including locally enriched motif clusters as defined in this study. Methods such as Argos [21] and Cis-Analyst [22] applied window-based strategies to detect local motif enrichment. Subsequent tools including MCAST [23], Cluster-Buster [24], MSCAN [25], TFBSCluster [26], and Stubb [27] expanded cluster detection capabilities, often supporting user-defined flexible window sizes to capture different binding signals. More recent approaches such as IDBC incorporated buffer and mutation concepts [28], while visualization-focused websites like TFmotifView facilitate motif display and exploration [29]. MCAST applies a Hidden Markov Model framework, is actively maintained within the MEME package [23], and supports integration with DNase I data. Many of these tools are not open source and no longer actively applicable (S1 Table) for our testing cases. Nevertheless, their published use cases provided valuable TF clusters for our evaluation. Building on these prior efforts, Motif-Cluster introduces a more flexible and open-source framework.

When it comes to reviewing the technical details, clustering motif matching sites along a linear genome naturally refers to spatial density-based methods. DBSCAN (Density-Based Spatial Clustering of Applications with Noise) [30,31] is a widely used algorithm that separates dense regions from background noise and has been implemented in libraries such as scikit-learn [32–34]. However, standard DBSCAN requires a pre-determined radius (epsilon: ε) and a threshold for the number of data points (min_samples) for the neighborhood, which may not be well suited for genomic data where inter-motif-site gaps vary across regions. HDBSCAN extends this framework by allowing hierarchical variation in ε, enabling detection of clusters at multiple density scales [30,31], but it does not explicitly incorporate signal weights such as binding affinity. OPTICS similarly relaxes fixed-density assumptions by identifying density-reachable structures [35], yet it also treats data points uniformly. In motif clustering applications, where binding sites differ in predicted affinity and gap distributions are heterogeneous across genomic contexts, incorporating both variable spacing and weighted signal strength is essential, motivating methodological adaptation beyond standard density-based implementations.

Accordingly, we developed Motif-Cluster, a computational framework that extends DBSCAN to incorporate both motif binding affinity (modeled as peak intensity) and adaptive gap parameters (ε), enabling dynamic detection of homogeneous TF binding clusters across genome. Beyond clustering, Motif-Cluster prioritizes and visualizes putative regulatory regions by integrating posterior scores derived from binding affinities and the likelihood of cluster gaps, providing a unified framework for cluster identification and regulatory ranking.

## Materials and methods

### Overview of Motif-Cluster

**Fig 1:**
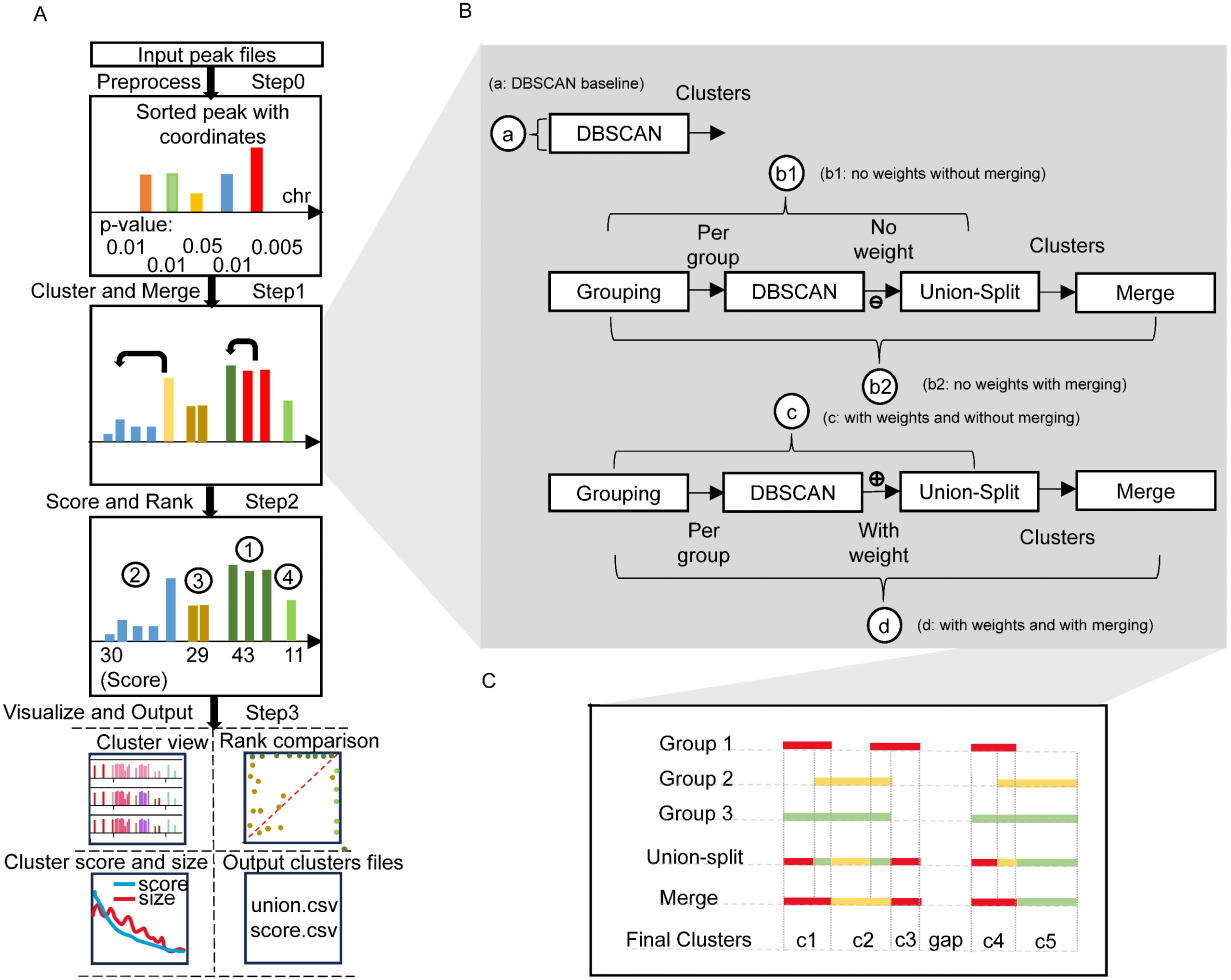
Motif-Cluster workflow and clustering strategies. (A) Overview of the Motif-Cluster pipeline, including preprocessing of motif peak files (Step 0), clustering and merging (Step 1), scoring and ranking (Step 2), and visualization and output (Step 3). (B) Comparison of clustering strategies evaluated in this study: (a: standard DBSCAN baseline; b1: gap-group-based method without affinity weighting or merging; b2: gap-group-based method with merging but without affinity weighting; c: gap-group-based method with affinity weighting but without merging; and d: the full Motif-Cluster framework incorporating gap grouping, affinity weighting, union-split refinement, and merging). (C) Schematic illustration of the grouping, union-split, and merge procedures. Binding sites are first clustered independently within different gap groups, followed by union-split refinement to resolve overlaps among groups and a final merging step to generate the reported motif clusters. The left and right portions illustrate consecutive examples within the same genomic region, separated by an intervening gap.

Motif-Cluster employs a modified DBSCAN algorithm to detect and prioritize local repetitive motif patterns. The methodology comprises four distinct steps for comprehensive analysis (Fig 1A). In Step 0, preprocessing, motif binding site data are prepared and sorted for subsequent analyses. Step 1, cluster and merge, involves the application of the modified DBSCAN algorithm to identify local motif clusters at genome wide scale. This step plays a crucial role in uncovering the repetitive binding patterns that may have been previously overlooked. In Step 2, the identified regions are subjected to a scoring and ranking process. This allows for the quantification of the significance and regulatory potential of each cluster, providing valuable insights into their repetitive binding patterns. Finally, in Step 3, which is optional, Motif-Cluster offers powerful visualization tools and generates informative outputs, enabling researchers to intuitively explore and interpret the prioritized transcription factor regulatory regions. This comprehensive approach ensures a robust and detailed analysis of motif binding sites, greatly enhancing our understanding of transcriptional regulation without any wet-lab experiments.

Motif-Cluster re-used several parameters and terms from DBSCAN and further adapted or re-defined them based on the needs of motif cluster analysis. Briefly, epsilon ε represents the neighborhood radius for grouping nearby binding sites; “min_samples” defines the minimum number of binding sites or total cluster weight required to form a cluster, which is the same as the minimum cluster intensity. Motif-Cluster invented “gap groups” to decompose the nucleotide distances between binding sites in the cluster to represent possible binding-site spacing patterns, refer to Gaussian-mixture-derived models. These are described in more detail in later sections.

### Data preprocess

Motif-Cluster processes TF binding files generated by FIMO [19] for genome-wide or chromosome-wide motif scanning, to generate a sorted bed file for further analysis. Alternatively, users have the flexibility to prepare TF scanning files using other tools and sort them using BEDTools [36]. The input bed file for subsequent steps must include well-organized information such as sorted chromosome start and end positions, along with either the p-value indicating motif matching significance or equivalent scores denoting binding affinity.

In this paper, the motif binding bed files are all downloaded via the PWMScan website [20], except that the PHB1 example is from FIMO scanning process. The selection of chromosomes and genome assemblies followed the original publications to facilitate direct comparison with previously reported regulatory regions. Specifically, motif binding tracks were downloaded from PWMScan using the JASPAR Core 2020 database with a p-value cutoff of 0.001. The datasets include ZNF410 human from chromosome 12 of hg19, ZNF410 mouse from chromosome 6 of mm10, EGR1 from chromosome 7 of hg38, and TWIST1 from chromosomes 6 and 8 of hg38. For the PHB1 analysis, we used the MEME Suite package (meme-5.5.1, February 4, 2023) together with the reported degenerate motif “RGYYYNRGYYY”. The motif was first converted into MEME format using the command “sites2meme motif> motif.meme”, followed by genome-wide motif scanning with FIMO using “fimo --oc. --verbosity 1 --thresh 0.001 motif.meme hg19.fa”. The resulting motif matches were then converted into BED format for downstream Motif-Cluster analysis.

### Cluster and merge

Motif-Cluster takes a sorted BED file as input and converts motif p-values into peak intensity scores using a negative log10 transformation. It then filters out binding-site gaps greater than 500 bp and constructs empirical gap distributions, allowing the analysis to focus on locally repetitive binding regions while excluding excessively long, likely non-regulatory intervals. We admit that the 500 bp gap cutoff lacks direct biochemical evidence; however, we suggest retaining it as a practical default threshold for separating locally coordinated motif clusters from widely spaced binding sites. A Gaussian Mixture Model (GMM) is applied to model the gap distribution, and the ten most supported Gaussian components are selected based on their means and variances. Increasing the number of components beyond ten does not substantially improve model fit as measured by AIC (Akaike Information Criterion) and BIC (Bayesian Information Criterion) [37,38] (S1 Fig). Therefore, we set ten as the maximum number of Gaussian components ($G_{i}$) for modeling gap distributions.

Next, Motif-Cluster performs density clustering based on the top ten gap groups, employing DBSCAN with varying parameters, according to the group-wise mean and standard deviation of the gap groups (Table 1). To loosen the criteria of neighborhood radius in DBSCAN, we use the mean plus twice the deviation $\mu \left(G_{i}\right)+\text{2}\sigma (G_{i})$ from each of the gap groups $G_{i}$ as the radiuses. This gap-group based DBSCAN process yields different clustered regions under each gap group. Motif-Cluster combines these ten groups of clustering (union) while also subdividing them into smaller clusters in cases of overlap (split). This dual approach ensures that the resulting clusters are finely tuned to account for different types of gaps. Given the presence of singletons and outliers, Motif-Cluster incorporates a final merging step (merge), allowing for the fusion of two nearby clusters that share a common, tolerated gap. The merging criterion is that the middle gap between two clusters or one cluster one singleton needs to be assigned to the same gap group as the nearby cluster based on the Gaussian component (the only exception is that the second probable gap group is also acceptable if the top probability is no more than 1.5 times the second probability). In ablation testing, Motif-Cluster explores different clustering strategies (Fig 1B and 1C), including direct DBSCAN without groups (method a), group-based DBSCAN with and without peak intensity score (between methods b1/b2 and c/d), and with or without merging (between methods b1/c and b2/d). Ultimately, only the method denoted as “d”, which employs both groupings and merging functions, emerges as the preferred and final methodology in Motif-Cluster.

**Table 1:** Internal parameters in testing cases for Motif-Cluster.

Method	ε (radius of neighborhood, same in DBSCAN)	Min_samples (number of samples or total weight, same in DBSCAN)	Motif_weight (binding affinity for single motif binding site)
a	G_mean_	8	10
b1	$\mu \left(G_{i}\right)+\text{2}\sigma (G_{i})$	8	10
b2	$\mu \left(G_{i}\right)+\text{2}\sigma (G_{i})$	8	10
c	$\mu \left(G_{i}\right)+\text{2}\sigma (G_{i})$	8	- log_10_(p-value)
d	$\mu \left(G_{i}\right)+\text{2}\sigma (G_{i})$	8	- log_10_(p-value)

G_mean_: average distance of all motif gaps; $\mu (G_{i})$: mean gap value from gap group i; $\sigma (G_{i})$: standard deviation of gap values from gap group i.

### Score and rank

Motif-Cluster takes the resulting clusters, representing putative regulatory regions with diverse binding site repetitive patterns, and conducts a scoring and ranking process based on their final scores. This score is derived from the posterior probabilities of both peak intensity and gap significance. Essentially, regions or clusters with more high-intensity peaks are given preference. However, large clusters with lower binding affinity can also hold significance. The gap size first must align with the most probable gaps identified by the Gaussian Mixture model [39], disfavoring atypical gaps that deviate from chromosome or genome-wide statistics, such as those that are overly long or excessively small. To achieve this, for each cluster, the mean gaps of the gap distances within the cluster will be used to calculate the best fitted gap group by the Gaussian Mixture model (formula (1)).


$$\begin{array}{c} i=\text{arg}{max}_{i}P\left(I_{mean}|C_{k},G_{i}\right) \end{array}$$ (1)

Where $I_{mean}$ is the mean binding affinity (peak intensity) for cluster $C_{k}$. The gap group for $C_{k}$ is therefore assigned to the most probable group $G_{i}$ by maximum likelihood.

Then for each of the gap groups, we build a group specific intensity distribution (empirical distribution $f(I|G_{i})$) to accommodate the varying contributions of the motif binding intensity given a specific group. Consequently, the final score of each region or cluster is computed as the cumulative sum of the log-likelihoods of the peak intensities and posterior gap probabilities (formula (2)).


$$\begin{array}{c} Score\left(C_{k}\right)=-\text{log}\left(P\left(C_{k}|I_{j},G_{i}\right)\right)=-\sum_{j}\text{log}(P(I_{j}|G_{i})) \end{array}$$ (2)

where $C_{k}$ means motif cluster k assigned to gap group i; $P(I_{j}|G_{i})$ is empirical probability for all binding site intensity $I_{j}$ within the region or cluster $C_{k}$. We use this score to rank all motif clusters and prioritize the most significant regulatory regions by the top scores. This score function will work for both single strong binding site or a cluster of medium or low binding affinities with a clear repetitive pattern. The random low affinity binding site will yield relatively low scores.

### Visualize and output

Motif-Cluster offers a user-friendly interface for visualizing motif binding site clusters following the preceding analytical steps. The visualization functions are optional downstream utilities that can be applied after the main Motif-Cluster analysis. Users have the option to specify start and end coordinates for a region of interest, allowing for the generation of a distinctively colored cluster view. Besides, Motif-Cluster can generate plots displaying cluster scores and sizes in descending order of score magnitude. Moreover, the tool facilitates comparative analysis of the same genomic region across different methods or datasets, effectively discerning the conservation of highly ranked regions. The primary output of Motif-Cluster comprises two files: the cluster-union file, which delineates which peaks or binding sites belong to the same cluster, and the cluster-score file, presenting a ranked listing based on cluster scores.

To ensure reproducibility and transparency, Motif-Cluster includes all partial method variants used in the ablation analyses. In addition, it generates diagnostic plots for validating the Gaussian mixture gap distributions and the peak intensity distributions within each gap group.

### Simulation functionalities in Motif-Cluster

Motif-Cluster implements two complementary simulation modules for generating synthetic transcription factor binding site distributions for the purpose of cluster identification. The first mode directly simulates motif binding BED files that can be immediately used as input for Motif-Cluster. In this mode, the simulated BED file records binding positions, motif intervals, and binding intensity values according to user-defined cluster and gap distributions specified in a JSON configuration file. Because the BED file represents the output of a motif scanning process, users do not need to explicitly generate genomic sequences or motif instances. This lightweight simulation mode is intended for method development, parameter testing, and ablation analysis.

To support benchmarking of external tools that require genomic sequence input, Motif-Cluster also provides a second simulation mode called Genome File Simulation. In this framework, users provide a small sample BED file containing representative motif sequences and associated binding intensities. The program then assembles synthetic (pseudo) genomic sequences by inserting these motif sequences into randomly generated background DNA according to specified cluster structures and gap distributions. This functionality enables generation of synthetic FASTA genomes suitable for end-to-end motif analysis pipelines involving motif scanning followed by cluster detection.

In this study, we used both simulation modes. First, we generated BED file simulations using ZNF410 derived gap distributions for method ablation testing and optimization of the Motif-Cluster framework. Although the exact gap values are not essential, real ZNF410 data were used to provide biologically realistic distributions. We repeated this simulation twice. Second, to compare Motif-Cluster with external tools such as MCAST [23] and Cluster-Buster [24], we generated synthetic genomic sequences using ZNF410 and PHB1 motif binding site examples. In these simulations, binding site patterns were derived from real datasets, while the surrounding genomic sequences were randomly generated with embedded motif sites. These two simulation strategies are described separately in the Results section.

## Results

### Simulations for Motif-Cluster method ablation test

To evaluate the performance of Motif-Cluster in identifying motif binding site clusters, we designed a simulation framework that generates synthetic input data resembling real motif scanning outputs in bed format. Pseudo-genomic coordinates were generated along a linear region spanning positions 1–300,000, mimicking a chromosome-scale sequence. Within this region, motif binding sites were simulated based on ten distinct cluster types, each defined by gap patterns whose mean and variance were derived from real ZNF410 binding data (S2 Table). For each simulated cluster, a cluster type was first sampled from these Gaussian mixture-based gap groups, followed by the random selection of the number of binding sites per cluster, ranging from 1 to 20. Each binding site was assigned a random p-value within the range of 10^-9^ to 10^-3^, reflecting the binding affinity distribution observed in real ZNF410 motif scanning results. Inter-site gaps within clusters followed the selected Gaussian gap model, while gaps between clusters were sampled either from the same Gaussian groups or set to values greater than 500 bp to represent non-clustered regions. Binding site coordinates were generated as genomic intervals by extending fixed-width regions from randomly chosen central positions.

Using the simulation procedure described above, we obtained approximately 140 clusters. We first performed ablation tests by evaluating partial implementations of the algorithm and multiple method variants. Across all tests, the final version of Motif-Cluster (method d) consistently produced cluster distributions that most closely matched the ground-truth simulated clusters. In one of the visualized regions, the complete Motif-Cluster implementation (method d) clearly distinguishes four homogeneous binding clusters within the locus, demonstrating improved separation compared to partial or baseline approaches (S2 Fig).

Methods that did not incorporate binding intensity or minimum cluster size tended to generate an excessive number of small clusters, failing to capture biologically meaningful clusters composed of weak but repetitive binding sites. In contrast, incorporating multiple binding gap patterns together with the final merging step substantially improved cluster recovery, enabling the identification of reasonable and coherent clusters that better reflect the underlying simulated regulatory structures. We also evaluated the potential impact of the minimum total weight used as the cluster threshold in the program (by changing the min_samples parameter in the program). Although varying this parameter led to different outcomes, our tests indicate that “min_samples” of 14 achieves the optimal performance, while the default value of 8 remains broadly acceptable in identifying big but reasonable size clusters.

The simulation framework is fully configurable through a JSON configuration file, allowing users to flexibly adjust Gaussian distribution parameters, chromosomal region length, cluster size ranges, p-value intervals, and inter-cluster gap interval widths. This design enables the simulation to be adapted to diverse transcription factors, motif properties, and experimental scenarios.

### Rediscovery of ZNF410 binding clusters in human and mouse CHD4 promoters

**Fig 2:**
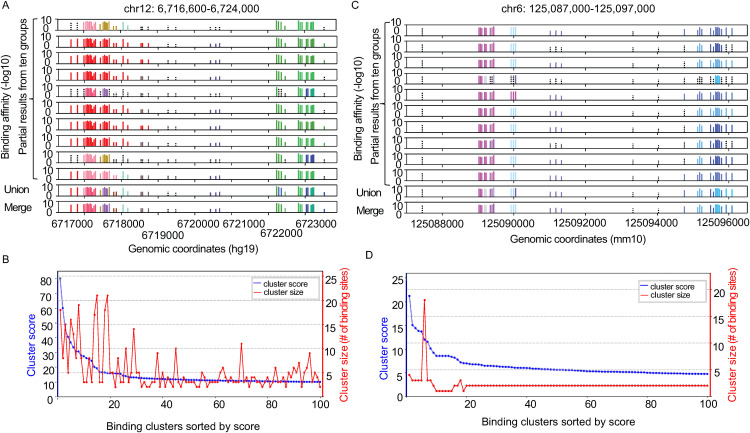
ZNF410 binding clusters at the CHD4 promoter in human and mouse. (A) Motif-Cluster results across the human CHD4 promoter region (chr12: 6,716,600-6,724,000; hg19), showing partial results and final clusters. **(B)** Cluster score (blue) and cluster size (red) for the top 100 ranked regions on human chromosome 12. **(C)** Motif-Cluster results across the mouse Chd4 promoter region (chr6: 125,087,000-125,097,000; mm10). **(D)** Cluster score (blue) and cluster size (red) for the top 100 ranked regions on mouse chromosome 6. Each color in the cluster panels represents an individual cluster.

**Fig 3:**
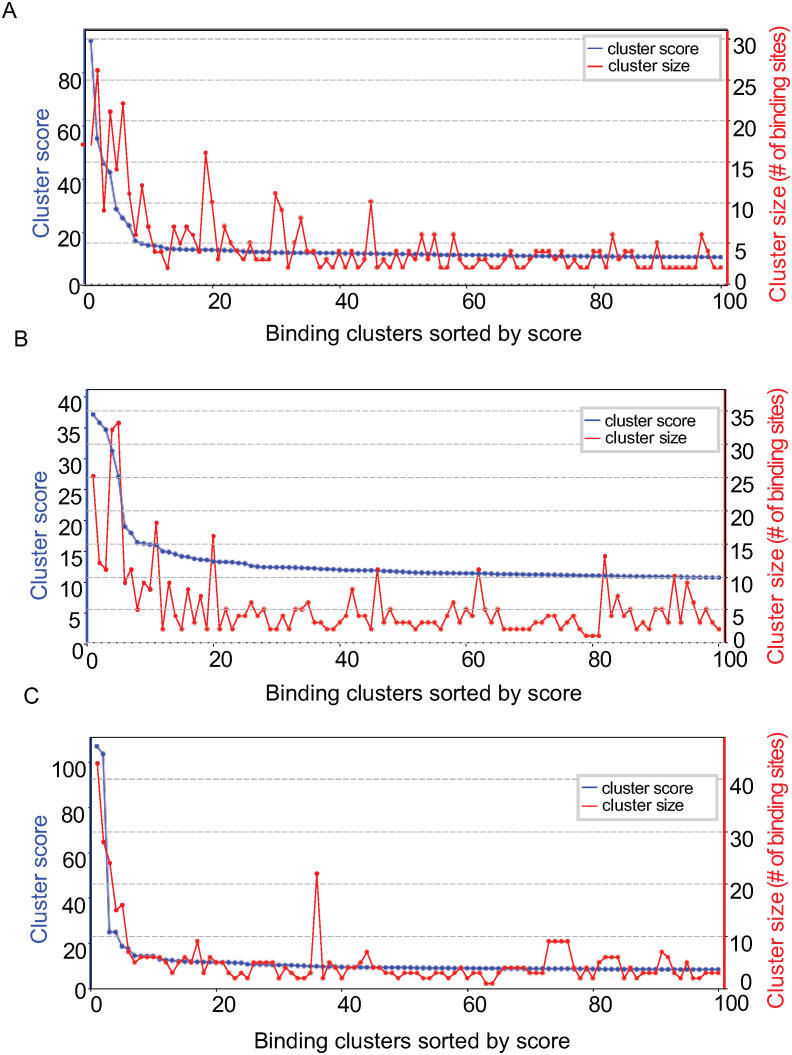
Top 100 prioritized ZNF410 binding clusters across human chromosomes. Cluster score (blue) and cluster size (red) are shown for the top 100 ranked ZNF410 motif clusters on (A) chromosome 11, (B) chromosome 6, and (C) chromosome Y (hg19). Clusters are ordered by decreasing score along the x-axis. The blue curve represents the integrated cluster score, and the red curve indicates cluster size.

ZNF410 is a recently identified key transcription factor involved in regulating fetal hemoglobin expression [13,14] and represents one of the few well-validated examples in which clustered binding sites define a functional regulatory region. Cut-and-run experiments have demonstrated conserved ZNF410 binding site clusters at the CHD4 promoter in both human and mouse genomes. Computationally, motif scanning reveals that ZNF410 motif matches are widespread across human chromosome 12 (hg19) and mouse chromosome 6 (mm10). Despite this pervasive distribution, Motif-Cluster successfully recovers the previously characterized CHD4 promoter cluster (Fig 2A and 2B). Although numerous putative binding sites are detected genome-wide, Motif-Cluster prioritizes the CHD4 region as the top-ranked regulatory cluster based on integrated cluster size and affinity scoring (Fig 2C and 2D; Table 2). Furthermore, Motif-Cluster can be readily applied to other chromosomes to prioritize candidate regulatory regions. We demonstrated this by analyzing human chromosomes 11, 6, and the Y chromosome without incorporating experimental binding data (Figs 3 and S3).

**Table 2:** Methods comparison in Motif-Cluster framework for ZNF410 cluster discovery.

Method	Description	Total number of clusters	Running time for step1	Running time for step2	Rank of the CHD4 promoter region
a	DBSCAN with neighborhood	153,758	1838 sec	382 sec	14
b1	No peak intensity, no cluster merge	210,797	6119 sec	856 sec	20
b2	No peak intensity, with cluster merge	139,379	7177 sec	798 sec	21
c	With peak intensity, no cluster merge	139,833	1558 sec	395 sec	8
d (Motif-Cluster)	With peak intensity, with cluster merge	58,606	1889 sec	395 sec	2

To further evaluate performance, we compared the full Motif-Cluster implementation (method “d”) with several variants, including the original DBSCAN approach (method “a”), versions with or without peak intensity weighting, and versions with or without the merging step (Fig 1). The complete Motif-Cluster framework produced fewer, more coherent clusters while maintaining a runtime comparable to standard DBSCAN. Importantly, the experimentally validated CHD4 promoter region ranked second among 58,606 clusters (Table 2), whereas none of the alternative methods placed this known regulatory region as highly. These results indicate that integrating peak intensity with group-based DBSCAN substantially improves prioritization of biologically meaningful clusters. It is noticeable that many potential clusters from this work do not have known functions yet. In addition, Motif-Cluster generated 46% of clusters with size three, whereas the alternative methods predominantly produced singletons (Table 3). This demonstrates that combining affinity weighting with gap-group modeling enhances detection of true binding site clusters rather than isolated motif occurrences.

**Table 3:** Number of clusters for different cluster size and their percentages using methods.

Cluster size / Method	a	a (%)	b1	b1 (%)	b2	b2 (%)	c	c (%)	d	d (%)
1	104990	68.283	195980	92.971	60053	43.086	95107	68.015	12257	20.914
2	32763	21.309	13559	6.432	71870	51.564	10432	7.460	11858	20.233
3	10751	6.992	1083	0.514	6676	4.790	27362	19.568	27443	46.826
4	3400	2.211	116	0.055	642	0.461	5895	4.216	5987	10.216
5	1158	0.753	23	0.011	89	0.064	790	0.565	807	1.377
6	413	0.269	5	0.002	10	0.007	174	0.124	177	0.302
7	144	0.094	5	0.002	9	0.006	31	0.022	32	0.055
8	58	0.038	7	0.003	10	0.007	15	0.011	17	0.029
9	23	0.015	4	0.002	4	0.003	10	0.007	10	0.017
10	9	0.006	3	0.001	4	0.003	4	0.003	5	0.009
>10	49	0.032	12	0.006	12	0.009	13	0.009	13	0.022
total	153758	100%	210797	100%	139379	100%	139833	100%	58606	100%

### Noise level tolerance testing with human ZNF410 binding sites

**Fig 4:**
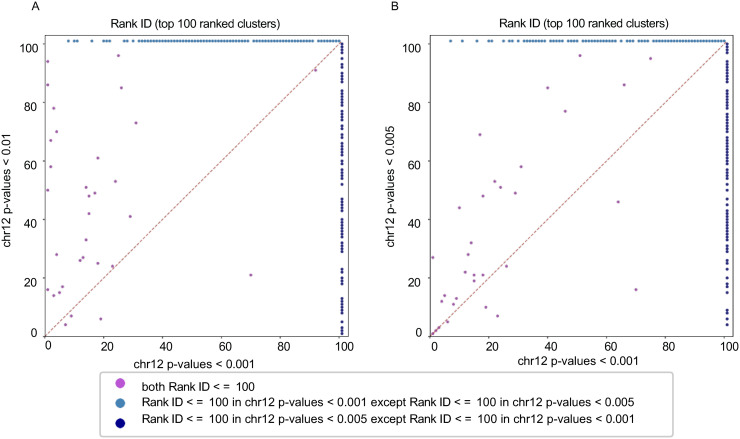
Comparison of top 100 ranked ZNF410 clusters under different motif binding affinity (p-value thresholds). (A) Rank comparison between clusters identified using stringent motif scanning (p-value < 0.001) and a relaxed threshold (p-value < 0.01) on human chromosome 12. (B) Rank comparison between clusters identified using p-value < 0.001 and p-value < 0.005 thresholds.

We generated ZNF410 binding sites with p-values p < 0.001 to represent high-affinity binding sites. However, determining the optimal p-value threshold for confident binding scores can be challenging for many transcription factors. Moreover, lower binding scores may play a functional role, particularly in the formation of larger clusters. We also created binding site lists with p < 0.005, and a high-noise version with p < 0.01. Upon examination (Fig 4), we find that the top 20 significant regions in the high-affinity result (p < 0.001) are well-preserved overall. Specifically, 7 out of the top 20 regions in p < 0.001 are retained in the p < 0.01 dataset, and 9 out of the top 20 regions in p < 0.001 are preserved in the p < 0.005 dataset. This indicates that while an increase in potential noise levels may have some impact, it is not overly significant, given that our Motif-Cluster method takes into account both binding affinity and cluster patterns. It is important to notice that these p-value thresholds are applied to the initial motif binding sites rather than the final cluster predictions. As additional motif sites or even noises are introduced under more relaxed thresholds, the gap distributions, cluster boundaries, union-split-merge operations, and cluster scores may all change, resulting in different cluster rankings. Therefore, the final top-ranked clusters are not expected to remain identical (or complete one-to-one preservation) across different motif-calling thresholds, even though the underlying motif sites from stricter thresholds (p < 0.001) are included within the more relaxed cases (p < 0.005 and p < 0.01). Nevertheless, the overlap among highly ranked clusters (such as top 20 identified clusters) suggests that Motif-Cluster is reasonably robust to moderate changes in motif-calling thresholds.

### Visualization at human CHD4 promoter region

**Fig 5:**
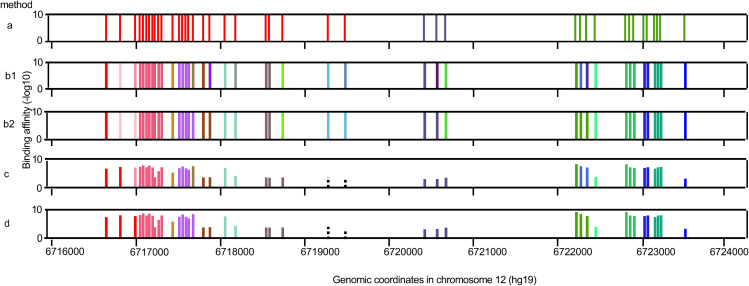
Comparison of clustering strategies for ZNF410 binding sites at the CHD4 promoter. Visualization of motif clustering results across the CHD4 promoter region (chr12:6,716,000-6,724,000) using different methods. From top to bottom: (a) standard DBSCAN with fixed neighborhood; (b1, b2) gap-based grouping followed by DBSCAN without affinity weighting; (c) grouping with merge; and (d) the full Motif-Cluster framework integrating gap grouping, peak intensity weighting, and merge. Colored bars represent binding sites grouped into clusters, with binding affinity shown as -log10(p-value). The full method as Motif-Cluster (d) produces more refined and coherent cluster separation compared with partial implementations.

We can now generate a comprehensive clustering comparison figure using the CHD4 promoter region (Fig 5). It is evident that when employing only DBSCAN (first row), there is an excessive clustering of certain binding sites that do not exhibit consistent gap ranges. However, with the incorporation of gap analysis, the other methods produce more refined clusters with comparable gap patterns. The third and fifth rows take merging into account, allowing for the elimination of some singletons and the expansion of clusters. The last two rows incorporate peak intensities, effectively removing low-affinity sites with insufficient cluster sizes. Ultimately, our Motif-Cluster favors the approach that considers both gap groups and peak intensity, prioritizing the refinement of clusters around chr12 6717000–6718000 and around 6723000, which is consistent with the previous studies [13,14]. Motif-Cluster supports other types of plots (see readme document of the software), such as score and rank curve, gap distributions, to help evaluate the clustering sites and calculations manually.

### More cases: PHB1, EGR1 and TWIST1 in human genome

**Fig 6:**
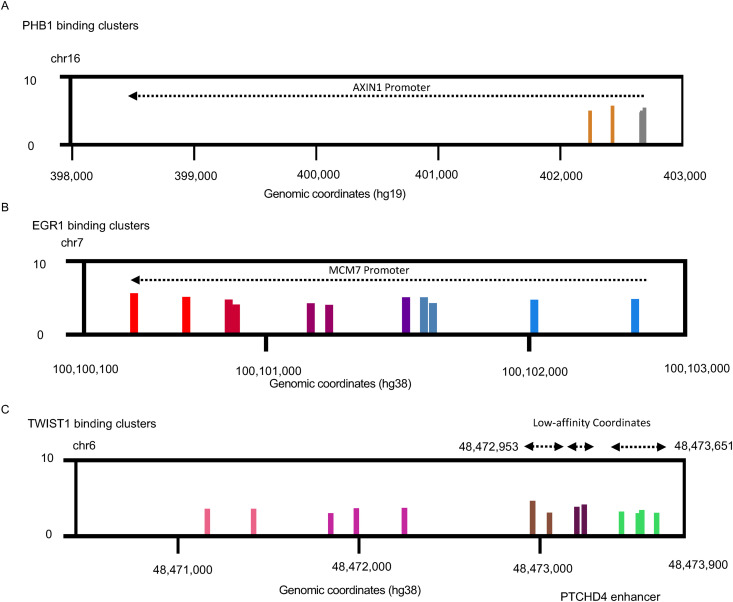
Motif-Cluster identification of validated regulatory binding clusters. (A) PHB1 clusters at the AXIN1 promoter. **(B)** EGR1 clusters at the MCM7 promoter. **(C)** TWIST1 clusters at the PTCHD4 enhancer. Colored bars indicate clustered motif binding sites.

PHB1 plays an important role in inhibiting Wnt/beta-catenin signaling through induction of Axin1 expression [40]. However, the PHB1 binding motif is highly degenerate and broadly distributed, making it challenging to pinpoint functional regulatory sites using motif scanning alone. Our approach addresses this challenge by identifying conserved binding clusters formed by degenerate motifs near regulatory regions. Specifically, we investigated the previously reported RGYYY motif (R = A/G, Y = C/T) by systematically searching for double RGYYYNRGYYY occurrences across the genome. Using FIMO on the human genome (hg19), we identified all candidate binding sites on chromosome 16, where AXIN1 is located. These regions were ranked based on motif repetitiveness and predicted binding affinity, and loci overlapping repetitive genomic elements were filtered to retain nontrivial candidates. Specifically, Motif-Cluster identified two significant clusters near the AXIN1 transcription start site (chr16:402657–402694; cluster size = 4;) (Fig 6A), consistent with previously reported regulatory regions whose functional relevance was validated by RNA-seq analysis [40]. The AXIN1 case study demonstrates that for highly degenerate motifs, where individual binding sites are often false positives, clustering substantially improves detection of biologically meaningful regulatory regions by enhancing robustness, improving ranking, and reducing single-site noise.

We further explored EGR1, a transcription factor shown in multiple studies to bind consecutively across promoter regions and influence gene regulation [7,28]. In the MCM7 promoter on chromosome 7, EGR1 shows a pattern of spatially clustered binding sites as well as isolated strong motifs, consistent with reports that such clusters can collectively contribute to regulatory activity. Using genome-wide motif scanning, Motif-Cluster identifies these adjacent EGR1 motif occurrences as a coherent cluster across the MCM7 promoter (Figs 6B and S4), highlighting regions of concentrated binding potential.

TWIST1 is a dosage-sensitive transcription factor with well-established roles in development and cancer, and previous work has shown that it directly regulates the PTCHD4 enhancer [8]. In the PTCHD4 locus on human chromosome 6, motif scanning reveals multiple, adjacent low-affinity TWIST1 binding sites spanning approximately chr6:48,472,953–48,473,651 (Fig 6C). Although individual motif matches are relatively weak, their close spatial arrangement suggests a clustered regulatory architecture. The detected motif clusters on chromosome 6 (chr6:48,472,983–48,473,484) and chromosome 8 (e.g., chr8:41,338,016–41,339,065 and chr8:123,232,060–123,232,824) are consistent with experimentally measured regulatory activity, as reflected by corresponding ATAC_ED50 and Luciferase_ED50 values (S3 Table). In both regions, clusters with higher Motif-Cluster scores align with loci showing functional enhancer activity, supporting the concordance between sequence-based clustering predictions and experimental validation. Using Motif-Cluster, these proximal TWIST1 motif occurrences are grouped into a coherent cluster, highlighting a region of concentrated binding potential that corresponds with the reported functional enhancer. This observation illustrates how aggregating weak motif signals into clusters can reveal biologically relevant regulatory elements that may be overlooked when considering single sites in isolation.

### Comparison with existing Motif Cluster tools

To further evaluate Motif-Cluster, we compared our method with two representative motif cluster analysis tools in command line mode: MCAST [23] from the MEME Suite (meme-5.5.9, November 22, 2025) and Cluster-Buster [24] (git commit: 06fee8b, September 27, 2024). Both tools are widely used for detecting locally enriched transcription factor motif regions from sequence data. Since neither MCAST nor Cluster-Buster directly accepts BED-formatted motif binding files as input, we extended the Motif-Cluster simulation framework to generate synthetic FASTA genomes together with embedded motif clusters for benchmarking purposes.

#### Simulation case A.

Using real ZNF410 binding sites from a BED file, we generated a synthetic genomic sequence of 300,000 bases containing 114 simulated motif clusters assembled from embedded binding sites. We then applied Motif-Cluster, Cluster-Buster, and MCAST using the same motif model (JASPAR Core 2020 MA0752.1) and the same synthetic FASTA genome. We attempted to optimize parameters across all methods to minimize differences introduced during motif calling; however, because Cluster-Buster and MCAST implement their own internal motif scanning and preprocessing strategies, the comparison may not be entirely equivalent. Under these conditions (S4 Table), Motif-Cluster achieved an average precision of 92.53% and recall of 84.95%, together with a motif calling rate of 99.92%. Cluster-Buster achieved the highest precision (97.03%) but substantially lower recall (47.53%) and a lower motif calling rate (26.69%), indicating a more conservative detection strategy. MCAST showed an average precision of 89.68% and recall of 33.33%, with a motif calling rate of 80.54%. Motif-Cluster generates cluster size distributions more similar to the simulated ground-truth distribution, achieving lower cosine distance (0.2344) and Euclidean distance (37.2028) compared with Cluster-Buster and MCAST.

#### Simulation case B.

We generated an additional simulation dataset using the degenerate PHB1 motif sequence “RGYYYNRGYYY” to represent a noisier and lower-affinity binding scenario compared with ZNF410. Using the provided PHB1 binding site sequences, we constructed a synthetic genomic sequence of 300,000 bases containing 131 simulated motif clusters. Motif-Cluster, Cluster-Buster, and MCAST were then applied to the same synthetic genome and motif inputs for comparison. In this setting, Motif-Cluster achieved an average precision of 96.14% and recall of 73.22% (S5 Table), while having all binding sites clustered. Cluster-Buster achieved lower precision (54.06%) and moderate recall (62.32%), whereas MCAST produced high precision (92.50%) but substantially lower recall (33.86%). Compared with the ZNF410 motif simulation, this dataset provides a fairer comparison because the simpler degenerate motif reduces discrepancies caused by motif scanning implementations, even though each tool still applies different internal motif-calling and cluster-detection strategies. Overall, Motif-Cluster maintained the best balance between precision and recall under this weak and noisy motif clustering scenario, demonstrating robustness in detecting biologically relevant motif clusters from degenerate sequence patterns. The lower cosine and Euclidean distances between Motif-Cluster predictions and the simulated cluster distribution demonstrate that Motif-Cluster more accurately recovers the expected motif cluster structures compared with Cluster-Buster and MCAST.

#### Znf410 case study.

we applied Cluster-Buster and MCAST to human chromosome 12 (hg19) using the ZNF410 motif MA0752.1 and compared the resulting motif clusters with Motif-Cluster (S5 Fig). Since Cluster-Buster identified 2,295 clusters, we summarized the cluster size distributions for the top 2,295 clusters across all three methods (S5A Fig). Although no genome-wide ground truth exists for all predicted ZNF410 clusters, several clear differences were observed among the methods. Motif-Cluster identified clusters spanning a broad range of cluster sizes, indicating its ability to capture both compact and extended motif cluster structures. MCAST also produced diverse cluster sizes, although with a stronger preference toward smaller clusters. In contrast, Cluster-Buster generated predominantly singleton clusters, with only a few larger clusters detected. To further compare the methods, we calculated the overlap among the top N ranked clusters between Motif-Cluster and the other tools (**S5B Fig**). Motif-Cluster and MCAST showed relatively consistent clustering behavior among the highest-ranked regions, particularly within the top 20 clusters, suggesting that both methods recover several common strong motif cluster signals. However, as more clusters were included, the overlap gradually diverged, indicating increasing methodological differences in lower-ranked predictions. By comparison, Cluster-Buster produced substantially different clustering results overall, with much lower overlap against Motif-Cluster across the ranked cluster sets.

## Discussion

The incorporation of both gap and affinity considerations in Motif-Cluster represents a significant advancement, as it obviates the need to exclude low-affinity binding sites. This is particularly useful, as even these low-affinity repetitive binding sites may harbor functional relevance, potentially acting as critical resources for transcription factor binding or indicating functionally significant genomic regions. This new approach broadens the scope of potential regulatory elements, enriching our understanding of transcriptional regulation. Many recent case-by-case studies on weak binding clusters further underscore the importance and necessity of our approach.

Motif-Cluster requires slightly more computational time for the cluster and merge step compared to the score and rank phase (S6 Fig). Future efforts should focus on optimizing the efficiency of these bottleneck functions within the clustering and merging processes by using BEDTools or pybedtools in a multi-process way [36,41]. Optimizing these operations by local dependency of the binding sites and conduct a genomic segment-wise parallel computing would further enhance the tool’s overall performance and expeditiousness.

One of the strengths of Motif-Cluster lies in its reliance solely on sequence data, without the need for experimental support. This is advantageous, particularly for transcription factors where conducting ChIP-seq experiments may be challenging, either due to antibody compatibility issues or the absence of a reliable global binding assay. Motif-Cluster’s robustness against noise allows for the utilization of even simple or degenerated motifs to generate an initial set of binding sites, subsequently enabling the prioritization of regulatory regions. While this stands as a significant advantage, future integration with experimental data, such as ChIP-seq [11,42] (and ChIP-seq databases such as GTRD [43] and Cistrome [44,45]), DNase-seq [45–53], and cut and run (cut and tag) assays [54–56], holds the potential to refine region prioritization; but we are not trying to re-develop an enrichment tool for omics experiments in this study. As a future interesting data resource, predicted motif clusters from Motif-Cluster can be intersected or co-displayed with experimental datasets mentioned above to further annotate TF occupancy evidence.

Importantly, Motif-Cluster is not limited solely to the prioritization of clusters with low affinities. It is also identifying crucial regions with single or few high-affinity binding sites, or a mixture of high or low affinity sites. This versatility ensures that the method remains applicable across a wide range of binding scenarios, underscoring its robustness and effectiveness in uncovering transcription factor regulatory regions, regardless of binding affinity. For parameter selection from the user’s perspective, we recommend beginning with the default settings (such as choice of minimum cluster intensity), as the method generally shows stable performance across a reasonable range of parameter choices. Users working with shorter or highly degenerate motifs may consider using more stringent thresholds to improve specificity, whereas more relaxed settings may help recover weaker but potentially cooperative motif binding clusters.

Motif-Cluster also provides several useful utility functions, particularly its simulation modules, which represent another unique strength of the framework. The package supports both direct BED-file simulation and synthetic genome generation, enabling reproducible benchmarking and comparison with external motif clustering tools. Given the limited number of experimentally validated motif clusters, the simulation functions provide a practical way to evaluate clustering behavior across diverse motif architectures, including weak and degenerate motifs. Currently, Motif-Cluster supports one transcription factor at a time, while combinatorial multi-transcription-factor clustering analysis remains a direction for future work.

One limitation of this study is that only a small number of experimentally validated functional motif binding clusters are currently available, restricting large-scale validation of Motif-Cluster across many confirmed regulatory regions, except by the simulation. In addition, any potential false positives largely originate from motif scanning itself rather than from the clustering framework, as Motif-Cluster focuses on spatial organization and ranking; false binding sites are generally deprioritized through affinity and clustering criteria as outliers. Nevertheless, the strength of Motif-Cluster lies in its sequence-based design, which enables systematic exploration of motif clusters even in the absence of large-scale experimental datasets.

Another limitation of this study is the comparison with existing motif clustering tools. Only a limited number of published methods remain easily accessible and reproducible through command-line implementations. In addition, these tools use different internal motif binding site calling and preprocessing strategies, making it difficult to fully standardize the number and distribution of detected binding sites for fair downstream cluster comparison. Nevertheless, based on both simulation datasets and real ZNF410 analyses, we observed that Motif-Cluster generally produces clustering results comparable to MCAST, particularly among highly ranked regions, whereas Cluster-Buster tends to generate substantially different clustering patterns.

## Conclusion

By integrating density-based clustering with flexible gap modeling and affinity weighting, Motif-Cluster provides a practical framework for clustering and ranking transcription factor regulatory regions directly from sequence data. This enables computational researchers to systematically prioritize candidate regulatory clusters genome-wide, even in the absence of experimental binding assays. For experimental biologists, Motif-Cluster offers a focused strategy to identify high-confidence regions for validation, accelerating hypothesis generation and study design. Together, these capabilities make Motif-Cluster a valuable tool for discovering previously overlooked regulatory elements and advancing our understanding of transcriptional regulation.

## Supporting information

S1 Table Relevant tool information for motif cluster and visualization. (XLSX)

S2 Table Simulation parameters and results. (XLSX)

S3 Table TWIST1 cluster binding sites and their validated ATAC-ED50 and Luciferase-ED50 scores. (XLSX)

S4 Table Simulation on a synthetic genomic sequence for ZNF410 motif and results from Motif-Cluster, Cluster-Buster and MCAST. (XLSX)

S5 Table Simulation on a synthetic genomic sequence for PHB1 motif and results from Motif-Cluster, Cluster-Buster and MCAST. (XLSX)

S1 Fig Performance for Gaussian mixture distribution for identifying groups for motif binding gaps. (TIF)

S2 Fig Visualization within one of the genomic regions in the simulation data with different method options. (TIF)

S3 Fig Binding cluster patterns for ZNF410 in Human genome chr11. (TIF)

S4 Fig Binding cluster patterns for EGR1 in Human genome chr7. (TIF)

S5 Fig Running external motif clustering tools on ZNF410 motif and human genome chromosome 12. (A) Cluster size distribution from three tools outputs (B) Comparison of overlapped motif cluster counts among the top N clusters across tools.
(TIF)

S6 Fig Running time measurement for clustering and ranking functions. (TIF)

## References

[1] Suter DM. Transcription Factors and DNA Play Hide and Seek. Trends Cell Biol. 2020;30(6):491–500. doi: 10.1016/j.tcb.2020.03.003 32413318

[2] Velthuijs N, Meldal B, Geessinck Q, Porras P, Medvedeva Y, Zubritskiy A, et al. Integration of transcription coregulator complexes with sequence-specific DNA-binding factor interactomes. Biochim Biophys Acta Gene Regul Mech. 2021;1864(10):194749. doi: 10.1016/j.bbagrm.2021.194749 34425241 PMC10359485

[3] Krieger G, Lupo O, Wittkopp P, Barkai N. Evolution of transcription factor binding through sequence variations and turnover of binding sites. Genome Res. 2022;32(6):1099–111. doi: 10.1101/gr.276715.122 35618416 PMC9248875

[4] Barnes SL, Belliveau NM, Ireland WT, Kinney JB, Phillips R. Mapping DNA sequence to transcription factor binding energy in vivo. PLoS Comput Biol. 2019;15(2):e1006226. doi: 10.1371/journal.pcbi.1006226 30716072 PMC6375646

[5] Kribelbauer JF, Rastogi C, Bussemaker HJ, Mann RS. Low-Affinity Binding Sites and the Transcription Factor Specificity Paradox in Eukaryotes. Annu Rev Cell Dev Biol. 2019;35:357–79. doi: 10.1146/annurev-cellbio-100617-062719 31283382 PMC6787930

[6] Maurano MT, Humbert R, Rynes E, Thurman RE, Haugen E, Wang H, et al. Systematic localization of common disease-associated variation in regulatory DNA. Science. 2012;337(6099):1190–5. doi: 10.1126/science.1222794 22955828 PMC3771521

[7] Khetan S, Carroll BS, Bulyk ML. Multiple overlapping binding sites determine transcription factor occupancy. Nature. 2025;646(8086):1001–11. doi: 10.1038/s41586-025-09472-3 40903577 PMC12447533

[8] Naqvi S, Kim S, Tabatabaee S, Pampari A, Kundaje A, Pritchard JK, et al. Transfer learning reveals sequence determinants of the quantitative response to transcription factor dosage. Cell Genom. 2025;5(3):100780. doi: 10.1016/j.xgen.2025.100780 40020686 PMC11960506

[9] Ulirsch JC, Nandakumar SK, Wang L, Giani FC, Zhang X, Rogov P, et al. Systematic Functional Dissection of Common Genetic Variation Affecting Red Blood Cell Traits. Cell. 2016;165(6):1530–45. doi: 10.1016/j.cell.2016.04.048 27259154 PMC4893171

[10] Melnikov A, Murugan A, Zhang X, Tesileanu T, Wang L, Rogov P, et al. Systematic dissection and optimization of inducible enhancers in human cells using a massively parallel reporter assay. Nat Biotechnol. 2012;30(3):271–7. doi: 10.1038/nbt.2137 22371084 PMC3297981

[11] Kidder BL, Hu G, Zhao K. ChIP-Seq: technical considerations for obtaining high-quality data. Nat Immunol. 2011;12(10):918–22. doi: 10.1038/ni.2117 21934668 PMC3541830

[12] Nordin A, Zambanini G, Pagella P, Cantù C. The CUT&RUN suspect list of problematic regions of the genome. Genome Biol. 2023;24(1):185. doi: 10.1186/s13059-023-03027-3 37563719 PMC10416431

[13] Vinjamur DS, Yao Q, Cole MA, McGuckin C, Ren C, Zeng J, et al. ZNF410 represses fetal globin by singular control of CHD4. Nat Genet. 2021;53(5):719–28. doi: 10.1038/s41588-021-00843-w 33859416 PMC8180380

[14] Lan X, Ren R, Feng R, Ly LC, Lan Y, Zhang Z, et al. ZNF410 Uniquely Activates the NuRD Component CHD4 to Silence Fetal Hemoglobin Expression. Mol Cell. 2021;81. doi: 10.1016/j.molcel.2020.11.006 PMC8556680 33301730

[15] Sher F, Hossain M, Seruggia D, Schoonenberg VAC, Yao Q, Cifani P, et al. Rational targeting of a NuRD subcomplex guided by comprehensive in situ mutagenesis. Nat Genet. 2019;51(7):1149–59. doi: 10.1038/s41588-019-0453-4 31253978 PMC6650275

[16] Vinjamur DS, Yao Q, Cole MA, McGuckin C, Ren C, Zeng J, et al. ZNF410 represses fetal globin by devoted control of CHD4/NuRD. Blood. 2020;136. doi: 10.1182/blood-2020-142134 PMC8180380 33859416

[17] Khan A, Fornes O, Stigliani A, Gheorghe M, Castro-Mondragon JA, van der Lee R, et al. JASPAR 2018: update of the open-access database of transcription factor binding profiles and its web framework. Nucleic Acids Res. 2018;46(D1):D260–6. doi: 10.1093/nar/gkx1126 29140473 PMC5753243

[18] Kulakovskiy IV, Vorontsov IE, Yevshin IS, Sharipov RN, Fedorova AD, Rumynskiy EI, et al. HOCOMOCO: towards a complete collection of transcription factor binding models for human and mouse via large-scale ChIP-Seq analysis. Nucleic Acids Res. 2018;46(D1):D252–9. doi: 10.1093/nar/gkx1106 29140464 PMC5753240

[19] Grant CE, Bailey TL, Noble WS. FIMO: scanning for occurrences of a given motif. Bioinformatics. 2011;27(7):1017–8. doi: 10.1093/bioinformatics/btr064 21330290 PMC3065696

[20] Ambrosini G, Groux R, Bucher P. PWMScan: a fast tool for scanning entire genomes with a position-specific weight matrix. Bioinformatics. 2018;34(14):2483–4. doi: 10.1093/bioinformatics/bty127 29514181 PMC6041753

[21] Rajewsky N, Vergassola M, Gaul U, Siggia ED. Computational detection of genomic cis-regulatory modules applied to body patterning in the early Drosophila embryo. BMC Bioinformatics. 2002;3:30. doi: 10.1186/1471-2105-3-30 12398796 PMC139975

[22] Berman BP, Nibu Y, Pfeiffer BD, Tomancak P, Celniker SE, Levine M, et al. Exploiting transcription factor binding site clustering to identify cis-regulatory modules involved in pattern formation in the Drosophila genome. Proc Natl Acad Sci U S A. 2002;99(2):757–62. doi: 10.1073/pnas.231608898 11805330 PMC117378

[23] Grant CE, Johnson J, Bailey TL, Noble WS. MCAST: scanning for cis-regulatory motif clusters. Bioinformatics. 2016;32(8):1217–9. doi: 10.1093/bioinformatics/btv750 26704599 PMC4907379

[24] Frith MC, Li MC, Weng Z. Cluster-Buster: Finding dense clusters of motifs in DNA sequences. Nucleic Acids Res. 2003;31(13):3666–8. doi: 10.1093/nar/gkg540 12824389 PMC168947

[25] Alkema WBL, Johansson O, Lagergren J, Wasserman WW. MSCAN: identification of functional clusters of transcription factor binding sites. Nucleic Acids Res. 2004;32(Web Server issue):W195–8. doi: 10.1093/nar/gkh387 15215379 PMC441525

[26] Donaldson IJ, Chapman M, Gottgens B. TFBScluster: a resource for the characterization of transcriptional regulatory networks. Bioinformatics. 2005;21:3058–9. doi: 10.1093/bioinformatics/bti461 15855248

[27] Sinha S, Liang Y, Siggia E. Stubb: a program for discovery and analysis of cis-regulatory modules. Nucleic Acids Res. 2006;34(Web Server issue):W555–9. doi: 10.1093/nar/gkl224 16845069 PMC1538799

[28] Lu R, Rogan PK. Transcription factor binding site clusters identify target genes with similar tissue-wide expression and buffer against mutations. F1000Res. 2018;7:1933. doi: 10.12688/f1000research.17363.2 31001412 PMC6464064

[29] Leporcq C, Spill Y, Balaramane D, Toussaint C, Weber M, Bardet AF. TFmotifView: a webserver for the visualization of transcription factor motifs in genomic regions. Nucleic Acids Res. 2020;48(W1):W208–17. doi: 10.1093/nar/gkaa252 32324215 PMC7319436

[30] Campello RJGB, Moulavi D, Sander J. Density-Based Clustering Based on Hierarchical Density Estimates. Lecture Notes in Computer Science. Springer Berlin Heidelberg. 2013:160–72. doi: 10.1007/978-3-642-37456-2_14

[31] Malzer C, Baum M. A Hybrid Approach To Hierarchical Density-based Cluster Selection. In: 2020 IEEE International Conference on Multisensor Fusion and Integration for Intelligent Systems (MFI), 2020. 223–8. 10.1109/mfi49285.2020.9235263

[32] Scikit-learn Documentation. scikit-learn.org. 2022.

[33] Varoquaux G, Buitinck L, Louppe G, Grisel O, Pedregosa F, Mueller A. Scikit-learn. GetMobile: Mobile Comp and Comm. 2015;19(1):29–33. doi: 10.1145/2786984.2786995

[34] Pedregosa F, Varoquaux G, Gramfort A, Michel V, Thirion B, Grisel O. Scikit-learn: Machine learning in Python. Journal of Machine Learning Research. 2011;12.

[35] Ankerst M, Breunig MM, Kriegel HP, Sander J. OPTICS: Ordering Points to Identify the Clustering Structure. SIGMOD Rec. 1999;28. doi: 10.1145/304181.304187

[36] Quinlan AR, Hall IM. BEDTools: a flexible suite of utilities for comparing genomic features. Bioinformatics. 2010;26(6):841–2. doi: 10.1093/bioinformatics/btq033 20110278 PMC2832824

[37] Kuha J. AIC and BIC: Comparisons of assumptions and performance. Sociological Methods and Research. 2004. doi: 10.1177/0049124103262065

[38] Burnham KP, Anderson DR. Multimodel inference: Understanding AIC and BIC in model selection. Sociological Methods and Research. 2004. doi: 10.1177/0049124104268644

[39] McCaw ZR, Aschard H, Julienne H. Fitting Gaussian mixture models on incomplete data. BMC Bioinformatics. 2022;23(1):208. doi: 10.1186/s12859-022-04740-9 35650523 PMC9158227

[40] Jackson DN, Alula KM, Delgado-Deida Y, Tabti R, Turner K, Wang X, et al. The Synthetic Small Molecule FL3 Combats Intestinal Tumorigenesis via Axin1-Mediated Inhibition of Wnt/β-Catenin Signaling. Cancer Res. 2020;80(17):3519–29. doi: 10.1158/0008-5472.CAN-20-0216 32665357 PMC7484901

[41] Dale RK, Pedersen BS, Quinlan AR. Pybedtools: a flexible Python library for manipulating genomic datasets and annotations. Bioinformatics. 2011;27(24):3423–4. doi: 10.1093/bioinformatics/btr539 21949271 PMC3232365

[42] Jiang S, Mortazavi A. Integrating ChIP-seq with other functional genomics data. Brief Funct Genomics. 2018;17(2):104–15. doi: 10.1093/bfgp/ely002 29579165 PMC5888983

[43] Yevshin I, Sharipov R, Valeev T, Kel A, Kolpakov F. GTRD: a database of transcription factor binding sites identified by ChIP-seq experiments. Nucleic Acids Res. 2017;45(D1):D61–7. doi: 10.1093/nar/gkw951 27924024 PMC5210645

[44] Mei S, Qin Q, Wu Q, Sun H, Zheng R, Zang C, et al. Cistrome Data Browser: a data portal for ChIP-Seq and chromatin accessibility data in human and mouse. Nucleic Acids Res. 2017;45(D1):D658–62. doi: 10.1093/nar/gkw983 27789702 PMC5210658

[45] Qin B, Zhou M, Ge Y, Taing L, Liu T, Wang Q, et al. CistromeMap: a knowledgebase and web server for ChIP-Seq and DNase-Seq studies in mouse and human. Bioinformatics. 2012;28(10):1411–2. doi: 10.1093/bioinformatics/bts157 22495751 PMC3348563

[46] Madrigal P, Krajewski P. Current bioinformatic approaches to identify DNase I hypersensitive sites and genomic footprints from DNase-seq data. Front Genet. 2012;3:230. doi: 10.3389/fgene.2012.00230 23118738 PMC3484326

[47] Sun H, Qin B, Liu T, Wang Q, Liu J, Wang J, et al. CistromeFinder for ChIP-seq and DNase-seq data reuse. Bioinformatics. 2013;29(10):1352–4. doi: 10.1093/bioinformatics/btt135 23508969 PMC3654708

[48] Koohy H, Down TA, Spivakov M, Hubbard T. A comparison of peak callers used for DNase-Seq data. PLoS One. 2014;9(5):e96303. doi: 10.1371/journal.pone.0096303 24810143 PMC4014496

[49] Moyerbrailean GA, Kalita CA, Harvey CT, Wen X, Luca F, Pique-Regi R. Which genetics variants in DNase-Seq footprints are more likely to alter binding? PLoS Genetics. 2016. doi: 10.1371/journal.pgen.1005875 PMC4764260 26901046

[50] Liu Y, Fu L, Kaufmann K, Chen D, Chen M. A practical guide for DNase-seq data analysis: from data management to common applications. Briefings in Bioinformatics. 2019. doi: 10.1093/bib/bby057 30010713

[51] Kähärä J, Lähdesmäki H. BinDNase: a discriminatory approach for transcription factor binding prediction using DNase I hypersensitivity data. Bioinformatics. 2015;31(17):2852–9. doi: 10.1093/bioinformatics/btv294 25957350

[52] Funk CC, Casella AM, Jung S, Richards MA, Rodriguez A, Shannon P, et al. Atlas of Transcription Factor Binding Sites from ENCODE DNase Hypersensitivity Data across 27 Tissue Types. Cell Rep. 2020;32(7):108029. doi: 10.1016/j.celrep.2020.108029 32814038 PMC7462736

[53] Yao Q, Ferragina P, Reshef Y, Lettre G, Bauer DE, Pinello L. Motif-Raptor: a cell type-specific and transcription factor centric approach for post-GWAS prioritization of causal regulators. Bioinformatics. 2021;37(15):2103–11. doi: 10.1093/bioinformatics/btab072 33532840 PMC11025460

[54] Meers MP, Bryson TD, Henikoff JG, Henikoff S. Improved CUT&RUN chromatin profiling tools. Elife. 2019;8:e46314. doi: 10.7554/eLife.46314 31232687 PMC6598765

[55] Can CUT&RUN and CUT&Tag replace ChIP-Seq? Genetic Engineering and Biotechnology News. 2020;40. doi: 10.1089/gen.40.12.09

[56] Akdogan-Ozdilek B, Duval KL, Meng FW, Murphy PJ, Goll MG. Identification of chromatin states during zebrafish gastrulation using CUT&RUN and CUT&Tag. Developmental Dynamics. 2022;251. doi: 10.1002/dvdy.430 PMC8976701 34647658

